# The association between polyphenol consumption and stroke risk factors in Saudi Arabia

**DOI:** 10.3389/fnut.2025.1696220

**Published:** 2026-01-06

**Authors:** Sarah N. Alsharif

**Affiliations:** Department of Clinical Nutrition, Faculty of Applied Medical Sciences, King Abdulaziz University, Jeddah, Saudi Arabia

**Keywords:** association, polyphenols, consumption, stroke, risk, Saudi

## Abstract

**Introduction:**

Eating foods rich in polyphenols has been shown in numerous trials to lower the incidence of stroke. The present study aimed to evaluate the relationship between stroke risk variables and the consumption of polyphenols.

**Methods:**

An online questionnaire was used to conduct a cross-sectional study involving 293 healthy persons aged at least 18 years in Saudi Arabia. Information was gathered on the demographics, health, use of supplements or drugs, smoking, anthropometric measures, physical activity, and dietary intake of six food products high in polyphenols.

**Results:**

The mean age of the participants was 34.82 ± 14.06 years, 72.7% were women, 51.5% were married, and 63.1% had a university education. Of them, 23.5% were obese and 62.8% were performing physical activity. Almost all participants had ≥3 modifiable stroke risk factors. Coffee was the most consumed polyphenol product (91.3%), followed by dates (88.7%) and apples (88.4%). Foods high in polyphenols were consumed anywhere from four to five times per day to less than once per month. The most commonly consumed polyphenol-rich foods were found in black tea, coffee, and apples. Except for milk chocolate, older participants consumed significantly greater amounts of polyphenol-containing food than younger ones. A higher intake of apples or dark chocolate was associated with a lower probability of having a stroke. Additionally, the risk of stroke is decreased by consuming more dark chocolate, cocoa powder, coffee, apples, black tea, and green tea.

**Discussion:**

Overall, adult Saudi Arabians who consumed more of a specific food high in polyphenols had a lower incidence of stroke. Additional long-term cohort studies is recommended to obtain more precise dietary data and to take into account randomized controlled trials to prove causation.

## 1 Introduction

Stroke is a common illness with rapidly developing symptoms that lead to either global or central brain impairment. Currently, it ranks as the second leading cause of death worldwide (1). According to Boehme and Esenwa (2), treatable risk factors include alcohol use, atrial cardiopathy, diabetes mellitus, hypertension, dyslipidemia, obesity, infection and inflammation, substance abuse, and smoking. Age, sex, race and ethnicity, and heredity are examples of untreatable risk factors.

Diabetes and hypertension are the most common traditional risk factors for stroke in Saudi Arabia (3). Furthermore, according to a Ministry of Health (MOH) study carried out in Saudi Arabia, the prevalence of stroke is 29 per 100,000 population per year (3). Polyphenols are secondary metabolites produced by plants and can be classified into stiblins, phenolic alcohols, phenolic acids, lignans, and flavonoids (4). Polyphenols provide a major protective role against various types of antibodies, including those that cause brain damage, atherosclerosis, stroke, cancer, and cardiovascular disease (CVD) (1). Table 1 provides an overview of the kinds and compositions of various foods high in polyphenols.

**Table 1:** Polyphenol types and content in selected foods.

Food item	Types of polyphenols	Polyphenol content/1 serving
Coffee	Caffeine, flavonoids, and phenolic acid from which (chlorogenic and caffeic) (54)	408 mg/190 g (55)
Oats	Sinapic acid, ferulic acid, and caffeic acid (56)	15.8–25.1 mg /40 g (56)
Apples	Phenolic acids (chlorogenic acid), anthocyanins (cyanidin), flavanols (catechin, epicatechin, procyanidins), flavonols (quercetin glycosides), and dihydrochalcones (phloretin glycosides) (57)	149 mg/110 g (55)
Dates	Anthocyanidins (quercetin, luteolin, apigenin, kaempferol), phenolic acids (gallic, protocatechuic, hydroxybenzoic, vanillic, isovanillic, syringic, caffeic, ferulic, sinapic, p-coumaric, and isoferulic) (58)	315 mg/21 g (3dates) (58)
Green tea	Catechins, serving as the base for 30 types of phenolic compounds, include epigallocatechin-3-gallate (EGCG), epicatechin (EC), epicatechin-3-gallate (ECG), and epigallocatechin (EGC)	173 mg/195 g (55)
Black tea		197 mg/195 g (55)
Cocoa chocolate	Epicatechins, anthocyanins, catechins, and procyanidins (59)	103 mg/3 g (55)
Milk chocolate		75 mg/32 g (55)
Dark chocolate		1,664 mg/100 g (55)

A study found that moderate consumption of coffee polyphenols (CPPs) was positively correlated with higher systolic and diastolic blood pressures (SBP and DBP) and hyperhomocysteinemia (5). Furthermore, it has been noted that excessive coffee drinking is believed to be a risk factor for CVD, whereas moderate coffee consumption has been observed to lower the risk (6). Consumption of phenolic compounds derived from oats demonstrated a considerable improvement in the 24-h DBP and SBP and reduction in the level of low-density lipoprotein (LDL) and cholesterol. Spencer et al. (7) suggest that phenolic compounds in oats are beneficial in preventing cardiovascular disease. Polyphenols are powerful epigenetic agents for cancer (8), and their administration can significantly reduce edema and cell damage after cerebral ischemia injury (9). Furthermore, various polyphenols may target particular long non-coding RNAs (lncRNAs), and the therapeutic effects of polyphenols may be mediated by important signaling pathways implicated in cancer intervention (10).

Date polyphenols have also been demonstrated to have anti-diabetic effects because they inhibit lipase, α-amylase, and pancreatic α-glucosidase, reduce lipid peroxidation, repair pancreatic cells, and limit the oxidation of free radicals (11). It has been demonstrated by numerous studies that polyphenols do, in fact, improve CVD risk factors. Foods high in polyphenols include cocoa, berries, and olive oil. A majority of these studies only take into account one source of polyphenol, the most basic forms of polyphenols, or specific diseases (12, 13). However, these studies do not address the ailments indicated in our study.

To our knowledge, this is the first cross-sectional study in Saudi Arabia to use an online questionnaire to assess adult male and female polyphenol consumption. Using a questionnaire, this study aimed to determine the quantity of polyphenols consumed through their diet by Saudi individuals in good health between January and March 2021. Moreover, a 3-month online survey conducted during COVID-19 was used to examine the relationship between Saudi adults’ intake of polyphenols and early-onset stroke risk variables.

## 2 Subjects and methods

### 2.1 Study design, setting, and time

A cross-sectional study was conducted in Saudi Arabia from January to March 2021.

### 2.2 Study participants

Men and women of Saudi nationality were initially recruited at the university hospital; however, due to COVID-19 regulations, we started recruiting data via an online Google Forms questionnaire. The inclusion criteria were healthy adults or those aged 18 years and above with at least one of the mentioned diseases (high blood pressure, diabetes, heart and blood vessel diseases, and high LDL cholesterol levels). Participants of other nationalities, those taking medications, patients with serious diseases affecting their condition, those who did not clearly state the amount of polyphenol intake needed, and finally, duplicated and repeated answers were being excluded from the study.

### 2.3 Sample size

The total sample size is 293, which was calculated using a *t*-test via G*power online software (Heinrich-Heine-Universität Düsseldorf, Germany) with 95% power, 5% significance, and 2% effect size.

### 2.4 Data collection

A validated, structured, electronic anonymous questionnaire was used with voluntary participants to complete questions with an explanation of answers before every question. Study participants were recruited by sending an online link to complete the questionnaire after filling out a consent form to be a part of the study, which includes information regarding the study. The duration of the data collection was 1 month.

A thorough, multi-step procedure was used to guarantee the questionnaire’s validity. To ensure content validity and cultural appropriateness, the questionnaire was first created in English and examined by a group of public health and nutrition specialists. Bilingual experts then translated it into Arabic, and to ensure correctness and consistency, the translation was then back-translated into English. To evaluate face validity, clarity, and comprehension, a pilot test was administered to a small group of individuals who were not part of the main study. Before the final administration, minor formatting and language changes were made in response to the comments. Together, these actions ensured the survey was legitimate, understandable, and appropriate for the target Saudi population’s culture. The questionnaire was divided into two sections: demographic data, which included age, gender, nationality, marital status, income, educational level, and employment status, and medical data, which included a history of specific diseases (hypertension, diabetes, heart and blood vessels diseases, and high LDL cholesterol levels), either a family history or the participants themselves, use of medications or supplements (vitamins and minerals), and whether smoking or not. Anthropometric measurements included weight and height and used them to calculate body mass index (BMI) to classify participants based on the number into either underweight (<18 kg/m^2^), normal weight (18–24.9 kg/m^2^), overweight (25–29.9 kg/m^2^), or obese (≥30 kg/m^2^). The physical activity level of participants was assessed according to duration and frequency using a questionnaire. Dietary intake assessment regarding six foods high in polyphenols (chocolate, oats, tea, apples, coffee, and dates) was assessed. The six polyphenol-rich foods included—coffee, black tea, green tea, apples, dates, and dark chocolate—were selected based on their high consumption prevalence in Saudi Arabia. For instance, one study found that 79% of study participants consumed Saudi coffee, whereas 69.3% consumed sweetened tea (14). Dates are a staple in the Saudi diet, and the consumption of dates reached 100 g/day, which could secure nearly 10th and 4th of the daily requirement of energy and non-starch polysaccharides, respectively (15). Dark chocolate has gained popularity in recent years, with over 50% of consumers preferring premium chocolate products. In addition, a previous Saudi study revealed that chocolate was the most frequently consumed snack by 70% of university students (16). A questionnaire was used to assess their usual intake, with every type of polyphenol having its own serving size explained to the participants briefly. The questionnaire was previously validated by two experts.

The primary study outcome was to assess the average consumption of the most polyphenol sources in the daily diet among Saudi adults, of both genders. In addition, it also assesses the association between commonly high polyphenol food sources consumed in Saudi Arabia (chocolate, oats, apples, tea, dates, and coffee) and early onset of stroke risk factors. The quantity of polyphenol intake was assessed based on self-reported frequency and amount of consumption of selected polyphenol-rich food items using a structured questionnaire. The polyphenol content of each food item was derived from the Phenol-Explorer database (17), which provides comprehensive data on polyphenol composition in foods. Standardized portion sizes were applied according to typical serving sizes commonly used in the Saudi population, as reported in national dietary surveys and local nutritional references. The secondary outcome was to examine the association between polyphenol consumption by binary categorization of the participants (any consumption vs. no consumption) for each food, with stroke risk factors. Predictor is specific to early onset of stroke risk factors (diabetes, hypertension, dyslipidemia, history of CVD, smoking, and obesity).

### 2.5 Ethical considerations

The Unit of Biomedical Ethics Research Committee of King Abdulaziz University in Jeddah, Saudi Arabia, granted ethical permission for the study (Protocol No. 55-21). Every procedure was carried out in compliance with the Declaration of Helsinki’s ethical guidelines. All replies were anonymized to preserve confidentiality, and participation was entirely voluntary. All respondents received information about the study’s goals, voluntary participation, and data confidentiality before their involvement. They were told that there would be no repercussions if they chose to leave at any point. The study team’s contact details were supplied in case there were any queries or issues. Informed consent was deemed to have been given upon completion and submission of the questionnaire.

### 2.6 Statistical analysis

The data were analyzed by Statistical Package for Social Science (SPSS) version 26.0 on IBM-compatible computer (SPSS Inc., Chicago, IL, United States). The qualitative data were described as numbers and percentages “*n* (%)” and analyzed using the chi-square test. Quantitative data were tested for normality using the Shapiro–Wilk test, assuming normality at *p* > 0.05. Quantitative data were described as mean, standard deviation, using Student’s *t*-test, if normally distributed, or the Mann–Whitney *U*-test, if not normally distributed. The accepted level of significance in this study was set at 0.05 (*p* < 0.05 was considered significant).

## 3 Results

This study enrolled 293 Saudi healthy individuals recruited at the university hospital and then via an online Google Forms questionnaire during the COVID-19 regulations. The mean age of the participants was 34.82 ± 14.06 years, and nearly half of them (48.5%) were younger than 30 years. Among participants, nearly three quarters (72.7%) were women, over half (51.5%) were married, nearly two-thirds (63.1%) had a university education, and 63.8% had a family income of more than 10,000. With regard to BMI, 69 (23.5%) subjects were obese. With regard to physical activity, 184 (62.8%) were performing physical activity, half of them performed physical activity two to four times per week, and more than one-third (37.5%) had physical activity for 21 to 40 min per day; 126 (43%) participants had supplementary vitamins and minerals such as iron, vitamin D, vitamin B, multivitamin, folic acid, vitamin C, omega 3, and zinc (Table 2).

**Table 2:** Baseline characteristics of the study group.

Total participants (*n* = 293)		
Gender	Men Women	80 (27.3%) 213 (72.7%)
Age (y)	34.82 ± 14.06	
Age groups	18–30 31–43 44–56 ≥57	142 (48.5%) 72 (24.6%) 54 (18.4%) 25 (8.5%)
Marital status	Single Married Widow Divorced	126 (43%) 151 (51.5%) 6 (2%) 10 (3.4%)
Kids (*N* = 145)	3.55 ± 1.07	
Education	Elementary Middle school High school Diploma University Master	1 (0.3%) 1 (0.3%) 52 (17.7%) 21 (7.2%) 185 (63.1%) 33 (11.3%)
Family income	<1,000 1,000–3,000 3,000–6,000 6,000–10,000 >100,000	5 (1.7%) 15 (5.1%) 28 (9.6%) 58 (19.8%) 187 (63.8%)
BMI	Underweight Normal Overweight Obese	7 (2.4%) 136 (46.4%) 81 (27.6%) 69 (23.5%)
Physical activity		184 (62.8%)
Frequency of physical activity per week (*n* = 184)	Once 2–4 times Daily	66 (35.9%) 92 (50%) 26 (14.1%)
Duration of physical activity per day (*n* = 184)	10–20 min 21–40 min 41–60 min ≥60 min	45 (24.5%) 69 (37.5%) 54 (29.3%) 16 (8.7%)
Supplements		126 (43%)

Data are presented as frequency (%) or mean (SD), BMI, body mass index.

**Figure 1:**
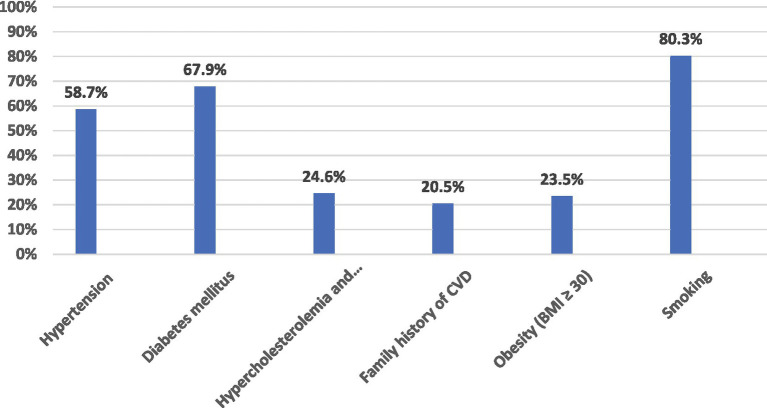
Distribution of modifiable stroke risk factors among the study group.

Almost all participants had three or more modifiable stroke risk factors (Figure 1) [either hypertension, diabetes, dyslipidemia, smoking, or obesity (BMI 30 kg/m^2^)]. Nearly 58.7% of the total group had hypertension, 67.9% had diabetes mellitus, 24.6% had dyslipidemia, and 80.3% were smokers (the most prevalent stroke risk factor recorded in the studied group).

With regard to the assessment of dietary intake of selected polyphenol food sources, coffee was the most consumed product (91.3%), followed by dates (88.7%) and apples (88.4%). The intake of food products of polyphenols ranged from less than once per month to four to five times per day, as illustrated in Table 3.

**Table 3:** Dietary intake assessment regarding selected polyphenol food products among the studied group.

Polyphenol food products	Total participants (*n* = 293)	
Dark chocolate	Does not eat <Once/month Once/month Once/week 2–4/week 5–6/week Once/day 2–3/day 4–5/day	106 (36.2%) 45 (15.4%) 37 (12.6%) 26 (8.9%) 22 (7.5%) 15 (5.1%) 31 (10.6%) 5 (1.7%) 6 (2%)
Milk chocolate	Does not eat <Once/month Once/month Once/week 2–4/week 5–6/week Once/day 2–3/day 4–5/day	36 (12.3%) 17 (5.8%) 37 (12.6%) 48 (16.4%) 54 (18.4%) 20 (6.8%) 58 (19.8%) 14 (4.8%) 9 (3.1%)
Cocoa powder	Does not eat <Once/month Once/month Once/week 2–4/week 5–6/week Once/day 4–5/day	128 (43.7%) 59 (20.1%) 31 (10.6%) 13 (4.4%) 18 (6.1%) 14 (4.8%) 25 (8.5%) 5 (1.7%)
Coffee	Does not drink <Once/month Once/month Once/week 2–4/week 5–6/week Once/day 2–3/day 4–5/day	25 (8.5%) 3 (1%) 13 (4.4%) 23 (7.8%) 30 (10.2%) 14 (4.8%) 92 (31.4%) 66 (22.5%) 27 (9.2%)
Oats	Does not eat <Once/month Once/month Once/week 2–4/week 5–6/week Once/day 2–3/day 4–5/day	89 (30.4%) 45 (15.4%) 35 (11.9%) 28 (9.6%) 24 (8.2%) 24 (8.2%) 34 (11.6%) 9 (3.1%) 5 (1.7%)
Apples	Does not eat <Once/month Once/month Once/week 2–4/week 5–6/week Once/day 2–3/day 4–5/day	34 (11.6%) 45 (15.4%) 51 (17.4%) 59 (20.1%) 28 (9.6%) 12 (4.1%) 51 (17.4%) 6 (2%) 7 (2.4%)
Dates	Does not eat <Once/month Once/month Once/week 2–4/week 5–6/week Once/day 2–3/day 4–5/day	33 (11.3%) 22 (7.5%) 30 (10.2%) 32 (10.9%) 27 (9.2%) 22 (7.5%) 75 (25.6%) 26 (8.9%) 26 (8.9%)
Black tea	Does not drink <Once/month Once/month Once/week 2–4/week 5–6/week Once/day 2–3/day 4–5/day	39 (13.3%) 16 (5.5%) 21 (7.2%) 26 (8.9%) 28 (9.6%) 19 (6.5%) 76 (25.9%) 50 (17.1%) 18 (6.1%)
Green tea	Does not drink <Once/month Once/month Once/week 2–4/week 5–6/week Once/day 2–3/day 4–5/day	86 (29.4%) 24 (8.2%) 19 (9.6%) 35 (11.9%) 32 (10.9%) 22 (7.5%) 47 (16%) 18 (6.1%) 10 (3.4%)

Data are presented as frequency (%).

Regarding the amount of food products of polyphenols, the largest amount consumed was black tea [mean (SD) = 273.43 (23.32)], followed by coffee [mean (SD) = 264.02 (21.25)] and then apples [mean (SD) = 156.84 (18.85)]. These findings are demonstrated in Table 4.

**Table 4:** Amount of intake (in grams) of 10 food products of polyphenols among the studied group.

Polyphenols food products	Total participants (*n* = 293)
Dark chocolate	11.59 (2.61)
Milk chocolate	47.66 (9.13)
Cocoa powder	6.97 (2.99)
Coffee	264.02 (21.25)
Oats	78.04 (9.77)
Apples	156.84 (18.85)
Dates	39.49 (12.09)
Black tea	273.43 (23.32)
Green tea	181.32 (24.53)

Data are presented as mean (SD).

On comparing the amount of intake of dietary products of polyphenols with regard to age, there was a significant difference between participants aged below 43 and those aged above 43, and with regard to the consumed amount of milk chocolate, apples, dates, black tea, and green tea, older participants consumed significantly higher amount of these products than younger ones, except for milk chocolate, in which case, younger participants consumed higher amount (Table 5).

**Table 5:** Mean consumption of 10 food products of polyphenols by age among the studied group.

Polyphenol food products	18–43 (*n* = 214)	44–57 (*n* = 79)	*p*-value
Dark chocolate	12.08 (2.59)	10.24 (2.49)	0.529
Milk chocolate	53.57 (9.67)	31.86 (6.76)	**0.033** ^a^
Cocoa powder	7.88 (2.69)	4.43 (1.83)	0.056
Coffee	260.12 (19.17)	274.43 (26.65)	0.176
Oats	78.24 (9.64)	77.52 (9.46)	0.954
Apples	138.84 (18.85)	204.94 (16.82)	**<0.001** ^a^
Dates	36.19 (3.13)	48.59 (3.23)	**0.003** ^a^
Black tea	234.82 (28.12)	380.41 (56.61)	**<0.001** ^a^
Green tea	158.49 (26.77)	247.57 (31.29)	**0.023** ^a^

Data are presented as mean (SD).

a Statistically significant. Bold values mean statistically significant.

Table 6 shows that the mean consumption of milk chocolate and cocoa powder was significantly higher among women compared to men, while the mean consumption of dates, black tea, and green tea was significantly lower among women (*p* ≤ 0.05). A non-significant gender difference was found according to dark chocolate, coffee, oats, and apples consumption (*p* ≥ 0.05).

**Table 6:** Mean consumption of 10 food products of polyphenols by sex among the studied group.

Polyphenol food products	Male (*n* = 80) mean (SD)	Female (*n* = 213) mean (SD)	*p*-value
Dark chocolate	13.42 (29.61)	10.89 (23.94)	0.717
Milk chocolate	36.73 (68.61)	51.68 (96.69)	**0.017** ^*^
Cocoa powder	1.23 (2.26)	9.07 (28.89)	**0.004** ^*^
Coffee	261.49 (250.23)	264.95 (200.05)	0.115
Oats	81.97 (98.24)	76.58 (95.03)	0.678
Apples	186.67 (177.52)	145.87 (86.00)	0.138
Dates	51.05 (40.50)	35.26 (27.30)	**0.001** ^*^
Black tea	356.76 (259.52)	243.35 (185.69)	**<0.001** ^*^
Green tea	271.58 (312.06)	149.64 (156.53)	**<0.001** ^*^

*means statistically significant. Bold values mean statistically significant.

Table 7 shows that underweight participants had a significantly higher mean consumption of dark chocolate, milk chocolate, and dates compared to other BMI categories (*p* ≤ 0.05), while obese participants had a significantly higher mean consumption of cocoa powder, coffee, and black tea (*p* ≤ 0.05). Overweight participants had a significantly higher mean consumption of green tea compared to other BMI categories (*p* ≤ 0.05), while there were no statistically significant differences according to oats or apples consumption (*p* ≥ 0.05).

**Table 7:** Mean consumption of 10 food products of polyphenols by BMI categories among the studied group.

Polyphenol food products	Under weight (*n* = 7) mean (SD)	Normal (*n* = 136) mean (SD)	Over weight (*n* = 81) mean (SD)	Obese (*n* = 69) mean (SD)	*p*-value
Dark chocolate	25.71 (36.90)	11.80 (25.32)	9.34 (24.56)	12.36 (26.15)	**0.027** ^*^
Milk chocolate	58.57 (51.70)	57.52 (79.29)	49.16 (128.36)	24.70 (44.70)	**<0.001** ^*^
Cocoa powder	4.57 (6.29)	4.18 (7.33)	9.25 (30.42)	10.25 (38.70)	**0.038** ^*^
Coffee	142.86 (133.63)	277.59 (194.02)	235.58 (178.87)	283.10 (284.25)	**0.030** ^*^
Oats	67.86 (96.52)	82.47 (100.33)	91.66 (98.67)	54.23 (79.10)	0.230
Apples	131.43 (72.39)	145.15 (80.14)	182.38 (118.49)	152.75 (174.00)	0.096
Dates	66.00 (23.76)	31.46 (21.26)	48.31 (45.38)	42.36 (27.23)	**<0.001** ^*^
Black tea	200.00 (173.21)	237.12 (177.83)	284.33 (215.83)	340.91 (259.94)	**0.046** ^*^
Green tea	35.71 (94.49)	161.40 (162.30)	216.33 (313.88)	198.81 (164.27)	**0.023** ^*^

*means statistically significant. Bold values mean statistically significant.

With regard to stroke risk factors and their relation to frequency of intake of food products of polyphenols, there was no statistically significant difference regarding intake of those products and the risk for each risk factors of stroke except for dark chocolate (the higher the frequency of dark chocolate intake, the lower the stroke risk, *p*-value 0.039) and apple (the higher the frequency of apple intake, the lower the stroke risk) (*p*-value 0.025) (Table 8).

**Table 8:** Association between consumption of polyphenol foods and the risk of stroke among the studied group.

Polyphenols food products	Diabetes mellitus (*n* = 199)	Hypertension (*n* = 172)	Dyslipidemia (*n* = 72)	History of CVD (*n* = 60)	Smoking (*n* = 192)	Obesity (BMI ≥30) (*n* = 69)	*p*-value
Dark chocolate	122 (61.3%)	103 (59.9%)	48 (66.7%)	37 (61.7%)	125 (65.1%)	56 (81.2%)	**0.039** ^*^
Milk chocolate	178 (89.4%)	152 (88.4%)	63 (87.5%)	57 (95%)	178 (92.7%)	56 (81.2%)	0.092
Cocoa powder	114 (57.3%)	96 (55.8%)	45 (62.5%)	38 (63.3%)	95 (49.48%)	39 (56.5%)	0.059
Coffee	181 (91%)	155 (90.1%)	62 (86.1%)	55 (91.7%)	175 (91.1%)	63 (91.3%)	0.117
Oats	150 (75.4%)	124 (72.1%)	53 (73.6%)	45 (75%)	125 (65.1%)	48 (69.6%)	0.067
Apples	179 (89.9%)	154 (89.5%)	65 (90.3%)	53 (88.3%)	166 (86.5%)	55 (79.7%)	**0.025** ^*^
Dates	182 (91.5%)	154 (89.5%)	68 (94.4%)	54 (90%)	163 (84.9%)	62 (89.9%)	0.213
Black tea	177 (88.9%)	149 (86.6%)	66 (91.7%)	57 (95%)	174 (90.6%)	67 (97.1%)	0.090
Green tea	149 (74.9%)	122 (70.9%)	48 (66.7%)	45 (75%)	144 (75%)	59 (85.5%)	0.210

Data are presented as numbers and frequency (%). *means statistically significant. Bold values mean statistically significant.

Regarding stroke risk and amount of consumed food products of polyphenols, the higher the amount of dark chocolate, cocoa powder, coffee, apples, black tea, and green tea intake, the lower the stroke risk (*p*-value 0.040, <0.001, <0.001, 0.021, 0.009, and <0.001), respectively. There was no significant difference between the amount of milk chocolate, oats, and dates and stroke risk (*p*-value 0.062, 0.067, and 0.055) (Table 9).

**Table 9:** Association between amount of consumed polyphenol food and the risk of stroke among the studied group.

Polyphenols food products	Diabetes mellitus (*n* = 199)	Hypertension (*n* = 172)	Dyslipidemia (*n* = 72)	History of CVD (*n* = 60)	Smoking (*n* = 192)	Obesity (BMI ≥30) (*n* = 69)	*p*-value
Dark chocolate	12.43 (2.67)	13.66 (2.85)	14.03 (2.73)	10.93 (2.18)	11.73 (2.64)	56 (8.2)	**0.040** ^*^
Milk chocolate	55.01 (9.8)	54.37 (10.28)	45.21 (7.25)	46.32 (7.63)	42.29 (7.26)	56 (8.2)	0.062
Cocoa powder	8.87 (2.99)	9.72 (3.2)	17 (4.37)	2.93 (4.7)	1.95 (0.48)	39 (5.65)	**<0.001** ^*^
Coffee	294.29 (18.38)	273.58 (23.96)	278.03 (25.1)	291.76 (25.89)	302.14 (26.97)	63 (9.3)	**<0.001** ^*^
Oats	81.15 (9.4)	73.78 (8.95)	83.92 (9.62)	75.85 (9.74)	66.94 (9.44)	48 (6.96)	0.067
Apples	161.29 (12.79)	164.8 (13.15)	157.42 (18.39)	191.29 (16.3)	157.29 (16.5)	55 (7.97)	**0.021** ^*^
Dates	39.87 (3.15)	37.48 (2.78)	43.39 (13.46)	39.42 (2.67)	45.78 (4.67)	62 (8.99)	0.055
Black tea	287.57 (21.58)	293.67 (22.13)	264.86 (19.99)	341.82 (26.22)	318.9 (24.68)	67 (9.71)	**0.009** ^*^
Green tea	20.83 (17.32)	199.55 (24.99)	216.9 (33.13)	208.33 (18.04)	214.76 (30.63)	59 (8.55)	**<0.001** ^*^

Data are presented as mean (SD). *means statistically significant. Bold values mean statistically significant.

## 4 Discussion

The current cross-sectional study was conducted in Saudi Arabia to assess the association between polyphenol consumption of selected food products and stroke risk factors.

The results of this study showed that, although coffee was the most consumed product among participants, black tea was the drink that contained the highest amount of polyphenols and was taken in greater quantities than coffee. This was consistent with other research conducted in Saudi Arabia, where the most popular commodity eaten was coffee (18, 19). Regarding research conducted abroad, a Japanese study discovered that the primary sources of polyphenols in their regular diet were green tea and coffee (20).

Our research showed that, except for milk chocolate, a higher intake of the majority of high-polyphenol foods was linked to an older age. This finding is in line with a prior study that involved 6,633 adults and found that subjects older than 70 years of age consumed more polyphenols overall than participants younger than that age (21). Older participants in the present study had a significantly higher mean consumption of apples, dates, black tea, and green tea. A previous Saudi study revealed the same result with a significant positive correlation between the total polyphenol consumption per gram and participants’ age (22). The mean consumption of milk chocolate and cocoa powder was significantly higher among women, which aligns with findings from previous Saudi studies (23, 24). One study found that frequent/weekly consumption of chocolate was more than 95% among girls (23). Another study found that daily candy/chocolate consumption was 52% among Saudi women adolescents compared to 37% among men (24). In contrast, the current study found that men had a significantly higher mean consumption of black tea and green tea compared to women. The same result was observed in a previous Saudi study, where sweetened tea consumption was significantly higher among men (25).

The present study revealed that underweight participants had a significantly higher mean consumption of dark chocolate, milk chocolate, and dates. Adults who consumed chocolate more frequently had a lower BMI than those who consumed chocolate less often (26). Consuming cocoa considerably reduced body weight, BMI, and waist circumference, according to a prior systematic review and meta-analysis (27). Higher frequency of chocolate consumption is associated with reduced BMI, which is consistent with an increasing amount of research indicating that both the kind and quantity of calories have an effect on metabolic equivalent of tasks (MetS) components (28, 29). There was a significantly higher mean consumption of cocoa powder, coffee, and black tea among obese participants and a significantly higher mean consumption of green tea among overweight participants in this study. This result disagrees with previous studies that validated the ability of tea and coffee and their components to reduce fat stores and body weight of humans (30, 31).

According to the current research, there is a substantial correlation between a lower risk of stroke and consuming more dark chocolate, cocoa powder, coffee, apples, black tea, and green tea. This result is in line with a previous randomized, placebo-controlled, double-blind trial that found that dark chocolate rich in polyphenols reduced blood pressure and fasting blood glucose in individuals with diabetes and hypertension, while also improving TG levels (32). Simultaneously, the results of the current investigation are consistent with those of a prior randomized, double-blind, placebo-controlled study involving 60 people with type 2 diabetes. According to the study, high-polyphenol dark chocolate successfully lowered blood pressure, fasting blood sugar (FBS), and lipid levels in hypertensive diabetic individuals (32). Additionally, ingesting 25 g of dark chocolate daily for 4 weeks resulted in a substantial decrease in both systolic and diastolic blood pressure as well as an increase in HDL cholesterol levels in another clinical research with 347 healthy Japanese volunteers (33).

Additionally, Morze et al. (34) found a marginally inverse correlation between the risk of stroke and each 10 g/day increase in chocolate intake. Furthermore, a more recent meta-analysis found that taking supplements containing 300–1,000 mg of cocoa per day from chocolate may provide modest protection against atherosclerosis, hypertension, diabetes, and stroke (35). Additionally, a substantial inverse relationship between chocolate consumption and the risk of having a stroke was observed in a large population-based prospective cohort study with a follow-up period of 12.9 years (36). Additionally, the relative risk (RR) of stroke was 0.81 for high chocolate consumption compared to low consumption, and 0.86 for every extra 50 g/week of chocolate intake, according to a meta-analysis of five prospective studies carried out in Europe and the US (37).

The underlying cause of this action may be the phytochemicals found in cocoa and dark chocolate, which have anti-inflammatory and antioxidant qualities and are related to nitric oxide regulation, which promotes vasodilation through activation and phosphorylation (38). Additionally, these phytochemicals stimulate antioxidant enzymes and lessen the generation of reactive oxygen species. The way in which they reduce inflammation is by modifying nuclear factor kappa B, which in turn reduces adhesion molecules within blood vessel walls and inflammatory markers, such as tumor necrosis factor-alpha, C-reactive protein, and pro-inflammatory cytokines. By decreasing the activation of adhesion molecules, pro-inflammatory cytokines, and nuclear factor κB (NF-κB) and by raising anti-inflammatory cytokines such as IL-10, the polyphenols in cocoa contribute to the slowing of the atherosclerotic process. They also assist in lowering glycated hemoglobin and fasting blood glucose, as well as improving lipid profiles by raising HDL and decreasing triglycerides and LDL. These substances also have antioxidant properties and affect a few hormones related to the reward system (38, 39). Cocoa polyphenols are known to have anti-inflammatory and antiplatelet effects, lower blood pressure, enhance metabolic health, and lessen atherosclerosis in addition to their antioxidant qualities. Additionally, they improve endothelial function, which raises the possibility that cocoa and its polyphenols have important cardioprotective advantages for people (39).

The results of the current investigation showed a strong correlation between consuming more black or green tea and a decreased risk of stroke. Our results are consistent with a prior study that found that drinking highly polyphenol-rich tea by older volunteers improved body weight, which is one of the stroke risk factors (21). Furthermore, a prior study with 487,377 Chinese adults discovered a correlation between a lower incidence of ischemic and hemorrhagic stroke and higher tea consumption, especially green tea (40). This is consistent with another cross-sectional study that found an inverse relationship between the prevalence of stroke in adult men in Korea and the use of green tea (41). In this regard, green tea considerably lowers the risk of stroke across a range of dosages and intake times, according to a recent systematic review and meta-analysis of 21 studies conducted in 2024 (42).

Our findings are in line with those of the Japan Collaborative Cohort Study, which involved 46,213 participants and discovered that coffee consumption can benefit both MI survivors and those without a history of MI or stroke (43). Green tea consumption may also improve the prognosis for stroke or MI survivors. An extra cup of tea per day is causally associated with a lower risk of small vessel stroke, according to research by Wang et al. (44) from 2021. Additionally, drinking green tea has been linked to a decreased risk of ischemic and cardiovascular disorders. Teas are a great source of polyphenols, and like flavonoids, they are known to lower blood pressure, cholesterol, blood glucose levels, and improve endothelial function (45).

According to our research, drinking more coffee was linked to a lower risk of stroke. This finding is in line with a different meta-analysis revealed a connection between coffee consumption and a lower risk of stroke, emphasizing coffee’s ability to prevent ischemic stroke in particular (46). Additionally, Miranda et al. (5) showed that moderate coffee consumption and its polyphenols were associated with a decreased risk of hyperhomocysteinemia and a lower likelihood of high systolic and diastolic blood pressure. Furthermore, a meta-analysis of 11 prospective studies found that those who drank six cups or less of coffee a day were less likely to suffer a stroke than people who did not, with comparable relative risks for ischemic and hemorrhagic stroke (6).

Despite the high prevalence of coffee consumption among participants, this study did not find a significant correlation between coffee consumption and stroke risk variables. There are a number of reasons behind this observation. Coffee varieties (e.g., Arabic, Turkish, or filtered), brewing techniques, serving sizes, and other additives (e.g., sugar and milk) that may change the overall polyphenol content and bioavailability were not differentiated in the questionnaire. Furthermore, prior research on coffee and cardiovascular health has produced conflicting findings. While some studies point to potential dangers associated with excessive drinking or high caffeine content, others demonstrate protective effects at moderate intake levels. Furthermore, the observed lack of correlation may have been impacted by unmeasured confounding factors such as smoking, stress, and general dietary habits. To elucidate the actual association between coffee consumption and stroke risk, future research involving a more thorough dietary evaluation and adjustment for these variables is advised.

In relation to apples, it was discovered that consuming more apples was linked to a lower risk of stroke. This result is consistent with a prior meta-analysis of eight pertinent randomized controlled studies, which discovered that eating apples and polyphenols generated from apples for more than a week can reduce levels of low-density lipoprotein (LDL) and total cholesterol (TC) (47). Additionally, taking 300 mg of apple polyphenols for 8 weeks improved vascular oxidative stress and endothelial function and decreased fasting plasma glucose and uric acid by inhibiting xanthine oxidase. This was demonstrated in a randomized, double-blind, parallel placebo-controlled clinical trial with 62 participants who had suboptimal fasting plasma glucose levels (48). According to eight randomized trials, eating 100 to 150 grams of whole apples a day was associated with a lower risk of cardiovascular disease, which is consistent with our findings. In addition, it raises high-density lipoprotein cholesterol and improves endothelial function while lowering blood pressure, pulse pressure, total cholesterol, low-density lipoprotein cholesterol, and inflammatory levels (49).

This study found a link between a lower risk of stroke and the high frequency of consumption of dark chocolate and apples, as well as the high amounts of dark chocolate, cocoa powder, coffee, apples, black tea, and green tea. This finding is in line with a cross-sectional study that involved 1,194 adolescent participants from Spain and discovered a negative correlation between total cholesterol (TC), diastolic blood pressure (DBP), and systolic blood pressure (SBP) in adolescents girls who consumed higher quartiles of total polyphenol intake. Furthermore, total polyphenol intake was found to be indirectly correlated with blood pressure, triglycerides (TG), low-density lipoprotein cholesterol (LDL-C), and high-density lipoprotein cholesterol (HDL-C) in boys and directly correlated with body composition and blood glucose, according to a structural equation model (50).

Due to their unique flavonoid content and greater absorption than other polyphenol sources, only apples and dark chocolate demonstrated a meaningful correlation with stroke risk variables. Quercetin in apples and flavanols in dark chocolate are well-known for their endothelial-protective and antioxidant qualities (51, 52). The lack of correlations with dates, tea, and coffee could be due to variations in serving quantities, preparation techniques, or additional additives such as milk and sugar that could lessen the bioactive potential of polyphenols. To elucidate the underlying mechanisms and validate these relationships in broader populations, future research should look more closely at these variances.

According to Lanuza et al. (53), consumption of total polyphenols, flavonoids, and phenolic acids was consistently and substantially associated with a lower risk of higher systolic blood pressure (SBP) and decreased levels of high-density lipoprotein cholesterol (HDL-C). Based on a structured questionnaire, the study’s findings indicate a correlation between the early onset of stroke risks and dietary intake of polyphenols from certain food sources. Since polyphenols have been found in a variety of food sources and have a great potential positive effect on health promotion in various levels of prevention, early protection would reduce damage, and as oxidative stress is the mediator for stroke-induced damage, this study may be helpful for future clinical trials that may be carried out in Saudi Arabia. This was the first study to evaluate the daily average consumption of the greatest sources of polyphenols among Saudi adults, both men and women, in Jeddah. Thus, if sufficient interventional research has been completed, a diet high in polyphenols should be suggested for therapeutic use. Additionally, studies on the bioactivity of polyphenols ought to be conducted *in vivo*, and *in vitro* studies on their mechanism of action are necessary in light of data supporting their efficacy (1).

This study’s main advantage is that it is the first to investigate any possible connections between stroke risk variables and high-polyphenol food consumption in Saudi Arabia. The study focuses on foods that are high in polyphenols that are typically consumed in Saudi Arabia, providing important health information to the general audience. Furthermore, a wide range of adult participants with different risk factors are included in the study, which improves statistical power to examine the association between polyphenol consumption and stroke risk. Additionally, study participants are probably going to comprehend and accurately record their nutritional intake because they are generally more health-conscious than non-participants. The literature on polyphenol products studied in Saudi Arabia is deficient, and currently, there is insufficient data or dietary reference intake addressing the intake of polyphenols for each class. The goal of this study is to close these gaps in knowledge by evaluating the consumption of the specified polyphenols among Saudis of various ages and reporting any correlations with risk factors for stroke.

Higher consumption of foods high in polyphenols, such as apples, coffee, and dark chocolate, was linked in our study to a decreased prevalence of stroke risk factors in Saudi adults. It is crucial to remember that our study is cross-sectional and can only describe associations, not causation, even though these results are consistent with earlier research indicating that polyphenols have cardiovascular benefits. Although they do not explicitly verify causal effects in our population, mechanistic studies and intervention trials—such as those that suggest cocoa polyphenols may enhance endothelial function or lessen oxidative stress—offer plausible explanations for these relationships. Therefore, such experimental evidence should be considered as information that generates hypotheses rather than as conclusive proof, even though it is useful for placing our results in context. To confirm these correlations and elucidate the underlying mechanisms, more longitudinal or interventional research is required.

### 4.1 Limitations

There are various restrictions on this study. First, it was difficult to determine a cause-and-effect link between the risk of stroke and the consumption of foods high in polyphenols due to the cross-sectional design. Second, residual confounding from other eating patterns, total energy intake, and similar variables may still affect the results due to missing information, even after correcting for multiple factors. Convenience sampling also has the ability to introduce bias into the research population’s representativeness. Furthermore, because of the study’s brief duration, long-term food patterns might not be adequately reflected. Self-reported data from volunteers were utilized to measure selected food intake; however, as self-reported questionnaires are frequently employed in large epidemiological research, this method may introduce errors. The COVID-19 pandemic’s restrictions on clinic access also constrained the study, and validation against gold standards. At the same time, we recognize that the fact that almost all participants had at least three modifiable stroke risk factors may indicate selection bias and restrict how broadly our results may be applied. This may be influenced by self-reported data, the online recruitment approach, and any lifestyle modifications brought on by the COVID-19 pandemic. We have addressed these issues and underlined the need for larger representative sample sizes in future research. Circulating polyphenol biomarkers might be helpful for future research. Moreover, the investigation did not take into consideration differences in the place of food origin, methods of preparation, or the addition of items to coffee, such as powdered creamer, sugar, or milk. As only six foods were chosen in our survey, we suggest that future research include a more thorough evaluation of polyphenol sources. This study’s failure to account for numerous comparisons when examining relationships between specific polyphenol sources and stroke risk variables was another limitation. This raises the possibility of type I error; therefore, care should be used when interpreting the strong correlations seen, especially between apples and dark chocolate. To confirm these results, future research should consider using multiple testing adjustments such as Bonferroni or false discovery rate (FDR). To further examine demographic and anthropometric variables such as age, sex, and BMI differences in polyphenol intake, future research with bigger and more varied populations should take into account stratified or multivariate analyses. Despite being gathered, information on cardiovascular illness in the family was not examined in connection with polyphenol intake. It is advised that larger and more varied sample sizes be used in future research to investigate whether people who are genetically predisposed to cardiovascular disease engage in more health-conscious eating habits, such as consuming more foods high in polyphenols. Finally, the online sample used in the study was chosen by self-selection, which could have resulted in sampling bias. Because of this, the observed prevalence of some risk factors, such as diabetes (67.9%) and smoking (80.3%), was greater, which might be due to sample discrepancies rather than actual population trends; therefore, the findings may not be representative of the Saudi population as a whole. Therefore, the results should be regarded cautiously, and more research using population-based or randomized sampling is advised to validate these findings.

In conclusion, this cross-sectional study discovered that Saudi individuals who consumed more of a few specific high-polyphenol foods had a lower risk of stroke. However, more extended cohort studies are required to validate these results. To prove causation, future studies should collect more precise dietary data and take randomized controlled trials into account. This study offers important insights into Saudis’ food consumption patterns related to polyphenols, despite its cross-sectional design. To ascertain whether the observed link is exclusively attributable to the chosen meals, more investigation is required.

## Data Availability

The original contributions presented in the study are included in the article/supplementary material, further inquiries can be directed to the corresponding author.
